# Resonantly enhanced multiple exciton generation through below-band-gap multi-photon absorption in perovskite nanocrystals

**DOI:** 10.1038/s41467-018-03965-8

**Published:** 2018-04-17

**Authors:** Aurora Manzi, Yu Tong, Julius Feucht, En-Ping Yao, Lakshminarayana Polavarapu, Alexander S. Urban, Jochen Feldmann

**Affiliations:** 1 Chair for Photonics and Optoelectronics, Department of Physics and Center for NanoScience (CeNS), Ludwig-Maximilians-Universität, Amalienstr. 54, 80799 Munich, Germany; 2 Nanosystems Initiative Munich (NIM), Schellingstr. 4, 80799 Munich, Germany

## Abstract

Multi-photon absorption and multiple exciton generation represent two separate strategies for enhancing the conversion efficiency of light into usable electric power. Targeting below-band-gap and above-band-gap energies, respectively, to date these processes have only been demonstrated independently. Here we report the combined interaction of both nonlinear processes in CsPbBr_3_ perovskite nanocrystals. We demonstrate nonlinear absorption over a wide range of below-band-gap excitation energies (0.5–0.8 *E*_g_). Interestingly, we discover high-order absorption processes, deviating from the typical two-photon absorption, at specific energetic positions. These energies are associated with a strong enhancement of the photoluminescence intensity by up to 10^5^. The analysis of the corresponding energy levels reveals that the observed phenomena can be ascribed to the resonant creation of multiple excitons via the absorption of multiple below-band-gap photons. This effect may open new pathways for the efficient conversion of optical energy, potentially also in other semiconducting materials.

## Abstract (web summary)

The effects of the combined interplay of below-band-gap multi-photon absorption and multiple exciton generation have not been investigated. Here, the authors show the combined interaction of these processes in perovskite nanocrystals, leading to an enhanced interband photoluminescence emission under resonant conditions.

## Introduction

Semiconductors are nowadays the most prominent materials for applications in optoelectronic devices. Harvesting the energy of light and converting it into usable electric energy with the lowest amount of losses is one of the main goals to achieve high efficiency in photovoltaic devices. The two main loss sources are (i) the absorption cutoff for photons with energies below the semiconductor band gap (*E*_g_) and (ii) the wasted excess energy of photons with energies above the band gap. Below-band-gap photons normally pass the respective semiconductor without being absorbed. Multiple photon excitation (MPE) processes, however, represent a route to access such low-energy photons leading to the generation of one exciton via the simultaneous absorption of a number (≥2) of below-band-gap photons whose combined energy is greater than the band-gap energy^1^. On the other side, highly energetic photons do not contribute effectively to the total usable energy in semiconducting devices because their excess energy is typically lost as heat. A possible approach to address this limitation is to generate multiple excitons from one photon, a process known as multiple exciton generation (MEG) or carrier multiplication^2–5^. This process typically requires photons with energies several times that of the band gap and has been shown to work efficiently only in nanocrystals exhibiting strong quantum confinement^6–12^.

**Fig. 1:**
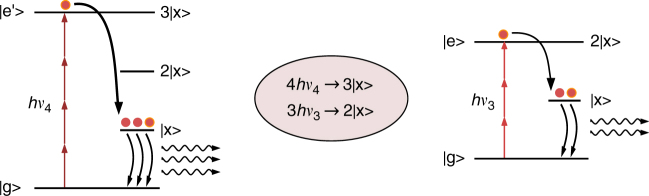
Schematic of the combined MPE-MEG mechanism. Multiple photon excitation with below-band-gap photons of energies *hν*_4_ and *hν*_3_ resonantly matching the energy levels 3|x> and 2|x> associated to multiple exciton generation

Here we investigate the combined action of MPE and MEG on the light-emitting properties of non-quantum-confined metal halide perovskite nanocrystals, a semiconducting material that in recent years has shown its high potential for photovoltaic^13,14^ as well as for light-emitting applications^15^. Although these mechanisms have been studied independently in perovskite nanocrystals^16–25^, here we analyze the possibility to combine them together to further enhance their efficiencies under specific resonant conditions. We find interband photoluminescence (PL) of CsPbBr_3_ nanocubes (NCs) under MPE for a wide spectral range below the band gap. Strikingly, we observe distinct resonances of the PL intensity for specific excitation wavelengths in the MPE-PL spectra. These correspond to specific energies of the excitation source and can be ascribed to the resonant creation of multiple excitons through MPE, as illustrated in Fig. 1.

## Results

### Synthesis and optical characterization

**Fig. 2:**
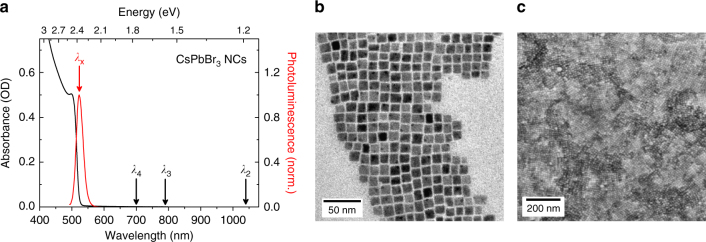
Optical and morphological characterization of the CsPbBr_3_ nanocubes. **a** Absorbance (left axis) spectra of CsPbBr_3_ nanocubes in hexane and normalized photoluminescence (right axis) of CsPbBr_3_ nanocubes on substrates, excitation wavelength 480 nm. The arrows indicate, respectively, the position of the photoluminescence emission *λ*_x_, and the excitation wavelengths *λ*_4_, *λ*_3_ and *λ*_2_ associated to the observed resonances. **b** TEM image of CsPbBr_3_ nanocubes, scale bar 50 nm. **c** SEM image of a CsPbBr_3_ nanocube film showing the superlattice-like arrangement, scale bar 200 nm

The CsPbBr_3_ NCs were synthesized using a one-step process based on direct tip sonication of precursor mixtures, according to our previously published work^26^. The purified dispersion of NCs exhibits an absorption spectrum characterized by a sharp absorption onset and a distinct excitonic peak at 500 nm (see Fig. 2a). The PL spectrum of the NCs deposited on a glass substrate shows a single PL emission peak centered around *λ*_x_ = 523 nm, corresponding to an energy *E*_x_ = *hν*_x_ = 2.37 eV. A spectrometer with an integrating sphere detector was used to measure the absorbance of the NCs deposited on a substrate (Supplementary Fig. 1). The absorbance of the NCs distributed on a glass substrate resembles the absorbance in solution, indicating almost no absorption of the nanocrystals for excitation below the band-gap. The average size of the cubes was determined from transmission electron microscopy (TEM) images to be in the range of 10–15 nm (Fig. 2b). These sizes along with the absorption and PL spectra clearly show that these NCs do not show quantum confinement, but have already bulk-like properties^27,28^. Moreover, from the TEM and the scanning electron microscopy (SEM) analysis (Fig. 2c), we observe that the NCs are mostly ordered on the substrate leading to a superlattice-like arrangement.

### Below-band-gap optical excitation

**Fig. 3:**
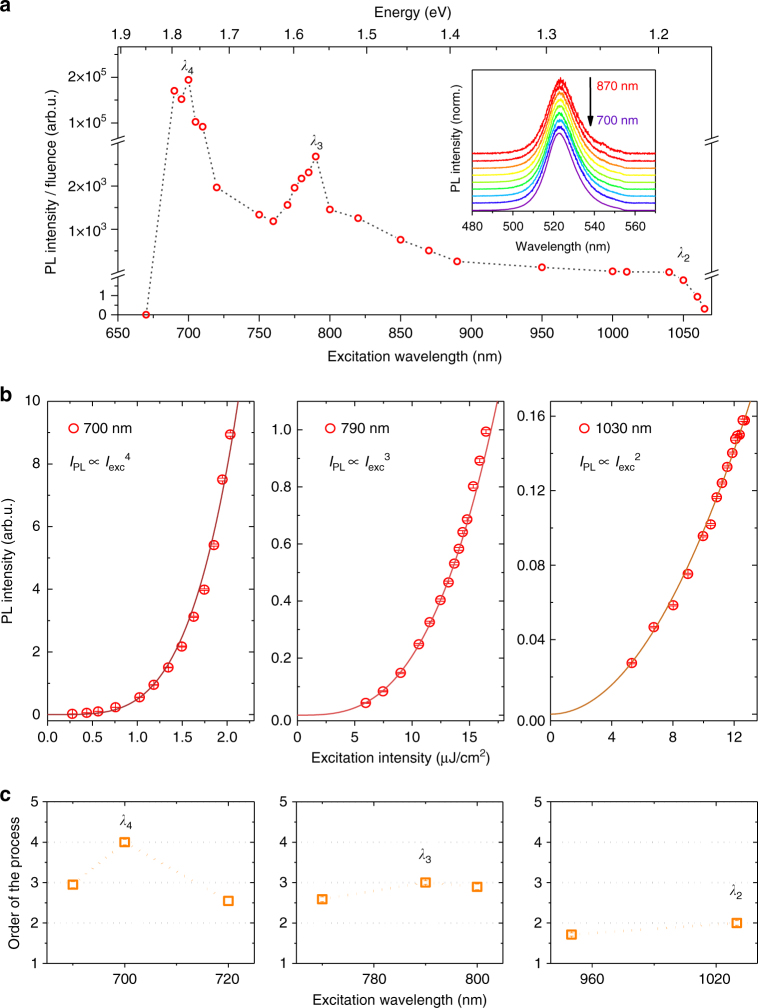
Nonlinear absorption-induced PL in perovskite NCs.** a** Photoluminescence intensity of CsPbBr_3_ NCs as a function of the below-band-gap excitation wavelength. The PL intensity is normalized by the laser fluence (see Supplementary Fig. 2). The dashed line is a guide to the eye. The inset shows the normalized photoluminescence spectrum for excitation wavelengths varying from 870 to 700 nm (specifically: 870, 850, 820, 800, 790, 770, 750, 720 and 700 nm). **b** Integrated photoluminescence intensity as a function of the laser excitation intensity for excitation wavelengths *λ*_4_ (700 nm), *λ*_3_ (790 nm) and *λ*_2_ (1030 nm). The solid lines represent the respective fit with power functions, from which we can deduce the order of the process: 4 for *λ*_4_ excitation, 3 for *λ*_3_ excitation and 2 for *λ*_2_ excitation. **c** Order of the absorption processes as a function of the below-band-gap excitation wavelength

By studying the emitting properties of the nanocrystals using an excitation source with below-band-gap energy photons, we observe that the CsPbBr_3_ NCs show PL emission for a wide range of excitation wavelengths (see inset in Fig. 3a). The excitation was provided by a focused supercontinuum laser (repetition rate 78 MHz) with 15 ps pulses tunable from 680 to 1080 nm. For each excitation energy we observe PL centered around 523 nm, indicating almost no shift with respect to the PL measured using above-band-gap excitation. Since the linear absorption of the NCs is negligible in this wavelength range, the absorption processes must be of nonlinear origin.

While the spectral shape of the PL emission does not change with wavelength, the PL intensity depends strongly on the excitation wavelength (see Fig. 3a). There is a clear onset of the PL intensity for an excitation wavelength around *λ*_2_ = 1030 nm (photon energy *hν*_2_ = 1.20 eV ≃ 0.50 *E*_g_), with the intensity generally increasing as the excitation wavelength is decreased. This onset can be associated to the minimum excitation energy required for two photons to overcome the band gap via MPE. A local maximum is reached at 680 nm, after which the PL intensity sharply drops to nearly zero. Even more interestingly, the MPE-PL spectrum does not increase monotonically, but rather exhibits two distinct peaks located at excitation wavelengths of *λ*_3_ = 790 nm and *λ*_4_ = 700 nm (photon energies *hν*_3_ = 1.57 eV ≃ 0.66 *E*_g_ and *hν*_4_ = 1.77 eV ≃ 0.75 *E*_g_, respectively). These peaks are associated with a PL intensity which is several orders of magnitude higher (10^3^ and 10^5^, respectively) than the signal detected in the vicinity of *λ*_2_. For clarity, the positions of the observed peaks in the MPE-PL spectrum are also marked with black arrows in the absorbance spectrum in Fig. 2.

Interestingly, these particular energies perfectly match the energy of multiples of the exciton energy *hν*_x_, suggesting the possible connection between multiple photon absorption and the resonant generation of multiple excitons. In fact, three photons of energy corresponding to the excitation wavelength *λ*_3_ have the exact energy required to generate two excitons in the NCs. In the same way, four photons of energy associated to the the excitation wavelength *λ*_4_ correspond to the energy necessary to generate three excitons. Analogously, two photons of energy related to the excitation wavelength *λ*_2_ exactly equal the exciton energy *E*_x_. No multiple exciton generation is therefore expected in correspondence to *λ*_2_, explaining the absence of an associated peak in the MPE-PL spectrum.

To exclude the possibility that the observed resonances are due to an effect only observable in CsPbBr_3_ NCs, we have performed analogous measurements on CsPbI_3_ NCs (Supplementary Fig. 3 and 4). These were fabricated through an ion exchange step from the originally synthesized CsPbBr_3_ NCs, preserving their size and geometry (see Supplementary Fig. 3 for more details). In these NCs, we again observe clear resonances in the PLE spectra. Their spectral positions again align with multiples of the exciton energy (*E*_x_ = 1.81 eV), i.e., they are red-shifted with respect to those observed in the CsPbBr_3_ system. These results support the notion that the proposed mechanism is not a specific property of CsPbBr_3_ NCs.

### Order of the absorption processes

For an absorption process involving *p* photons, the intensity of the PL, *I*_PL_, is proportional to the excitation intensity to the power of *p*, $(I_{{\mathrm{exc}}})^p$, with *p* being the order of the process. For *p* = 1 this accounts to the regular linear absorption, while for *p* > 1 the process corresponds to a multiple photon absorption process via *p*−1 virtual states. Thus, by measuring the PL intensity in dependence of the excitation intensity and applying a power law fit, one can obtain the order of the process and thus determine the nature of the absorption.

In Fig. 3b the integrated PL intensities at the respective excitation wavelengths *λ*_4_, *λ*_3_ and *λ*_2_ are plotted in dependence of the laser excitation intensity. Fitting power law functions to the data, we find that at *λ*_4_ absorption takes place via a fourth-order process, while at *λ*_3_ the process is of third order and at *λ*_2_ of second order. This is in complete accordance with our interpretation of an MPE process.

### Analysis of the energy levels

**Fig. 4:**
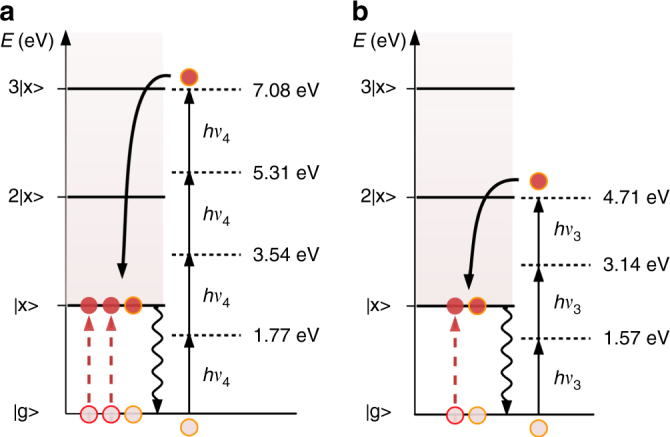
Energy diagram of the resonances between MPE and MEG in CsPbBr_3_ NCs. Photo-excitation at 3*E*_x_ (**a**) and 2*E*_x_ (**b**) and subsequent generation three (**a**) and two (**b**) excitons via multiple photon excitation processes with photons of energies *hν*_4_ and *hν*_3_, respectively

With the knowledge that 4 and 3 photons are involved in the absorption processes associated to the excitation wavelengths *λ*_4_ and *λ*_3_ respectively, we can determine the energy levels reached by the MPE process and correlate them with the energy necessary to generate multiple excitons. A schematic representation of the combined MPE and MEG processes in the analyzed system is shown in Fig. 4. When *λ*_4_ is used as the excitation wavelength, the system undergoes 4-photon absorption and reaches the energy level 7.08 eV, that is resonant with 3*E*_x_ = 7.10 eV. Similarly, when photons with wavelength *λ*_3_ are used, a 3-photon absorption process is observed and the photogenerated electrons have an energy of 4.71 eV, resonant with 2*E*_x_ = 4.73 eV. Therefore, the energy matching between the energy level reached through MPE and the level associated to multiples of the exciton energy *E*_x_ allows the combined interplay of MEG and MPE. The combination of these processes produces the observed maxima in the PL efficiency for specific values of the excitation energy, which is in agreement with the efficient creation of multiple excitons.

## Discussion

The observation of resonances given by MEG in the MPE-PL of CsPbBr_3_ is realized when the following relation is fulfilled:

1 $$p \times h\nu = N \times E_{\mathrm{x}}$$

where *p* is the number of photons involved in the MPE, *N* is the number of excitons created through MEG and *E*_x_ is the energy of a single exciton. In order to confirm this theory, we look at MPE at excitation wavelengths away from the observed resonances and focus on the order of the excitation process (Fig. 3c). The PL intensity drops off steeply for excitation wavelengths longer than 1030 nm, corresponding to twice the band-gap energy of the CsPbBr_3_ NCs. For slightly shorter wavelengths, the PL intensity scales proportionally to (*I*_exc_)^2^, while for slightly longer wavelengths the PL intensity clearly scales with (*I*_exc_)^3^ (Supplementary Fig. 5). These values confirm that the drop off region marks the onset of two-photon absorption (2PA), while PL emission for excitation at longer wavelengths requires three photon absorption (3PA). For shorter wavelengths the order of the absorption process remains around 2 or slightly below this value (see Supplementary Fig. 6). Close to the resonance at *λ*_3_ the order jumps up to a value of 3 again, and at *λ*_4_, the order increases to 4. In between these resonances, the order decreases rapidly to 2. This suggests that the specific resonances are indeed necessary to promote the higher order MPE. Notably, the PL decreases abruptly to almost zero for excitation wavelengths shorter than 680 nm. At these wavelengths, four photon absorption leads to energies in excess of 7 eV, which could possibly induce ionization of the NCs, opening a new non-radiative decay channel.

Upon closer inspection, the peaks in the PL spectrum corresponding to excitation wavelengths in the vicinity of resonances have a characteristic non-symmetrical shape (Fig. 3a). Surprisingly, when photons with energies slightly higher than the resonance energy are used for excitation, the efficiency of the emission drops. This is an indication that the combined MPE-MEG process is particularly favorable when no additional excess energy is involved in the excitation process.

Another striking result is that the resonant MPE-MEG process is absent for such nanocubes under comparable laser excitation intensities when they are dispersed in solution. This suggests that the observed PL enhancement in correspondence with the MEG resonances relies on the close-packed arrangement of the CsPbBr_3_ NCs on the substrate. As reported by Trinh et al., MEG in adjacent silicon nanocrystals results in a suppression of the non-radiative Auger recombination and in the creation of long-living multiple excitons^29^. The initial optical excitation possesses a large energy and the related wavefunctions can overlap spatially with those of adjacent nanocrystals, if they are close enough. The highly energetic excitation can then create multiple excitons in different adjacent nanocrystals. The spatial extent of these wavefunctions is much smaller, consequently Auger recombination becomes less likely and in turn radiative recombination is enhanced. This model thus offers an explanation for the radiative character of MEG in our system.

To substantiate our proposed model, we have also measured a highly diluted dispersion of CsPbBr_3_ NCs on substrate (see Supplementary Fig. 7). While we could measure linearly excited PL from these NCs, we did not observe the MPE-MEG resonances observed for a dense array of NCs. This further supports the idea that a dense arrangement of the NCs is crucial for observing this phenomenon and for suppressing non-radiative Auger recombination. Further validity is obtained by repeating the investigations on larger CsPbBr_3_ nanocubes with edge sizes on the order of 100 nm (Supplementary Fig. 8). While we observe an enhanced PL near the expected resonance positions, the resonances themselves—if present—are significantly weaker and strongly broadened. This is to be expected, as Auger recombination will not be similarly suppressed as in the case of the smaller close-packed NCs.

In conclusion, we have shown that perovskite NCs exhibit interband PL emission when excited with photons with below-band-gap energies over a wide range of energies. Our findings show that multiple excitons can be generated in non-quantum-confined perovskite NCs by the excitation with multiple photons and that the system exhibits resonances for specific values of the excitation wavelength. These resonances occur when the total energy of a number of exciting photons is equal to the energy necessary to create multiple excitons. Owing to the presence of resonances, the photogeneration together with the radiative recombination of excitons are highly enhanced, while the non-radiative Auger recombination is suppressed due to the spatial arrangement of the NCs on substrate. Therefore, high-order MPE can be used as an efficient pathway for the creation of multiple excitons in non-quantum-confined perovskite nanocrystals. Moreover, having observed the presence of MEG-MPE resonances in different perovskite NCs, we can envision this process also occurring in other semiconducting nanocrystals even with sizes greater than the quantum dot restriction. This could open new pathways for various energy conversion systems exploiting high light intensities.

## Methods

### Materials

The synthesis of CsPbBr_3_ NCs was carried out by ultrasonication of precursors in octadecene solvent mixed with oleylamine and oleic acid ligands, as reported previously by our group^26^. A detailed description of the synthesis techniques can be found in the Supplementary Note 1.

### Experimental setup

The excitation for the MPE-PL measurements was provided by a SuperK EXTREME supercontinuum white light laser focused by a 100× magnification to the specimen. More information about the experimental setup is in the Supplementary Note 2. All the measurements were conducted in normal atmosphere and at room temperature.

### Feasibility studies

An estimation of the efficiency of the proposed mechanism for solar applications can be found in the Supplementary Note 3.

### Data availability

The data that support the findings of this study are available from the corresponding authors on reasonable request.

## Electronic supplementary material

Supplementary Information
