# Supplementary material for: Resonantly enhanced multiple exciton generation through below-band-gap multi-photon absorption in perovskite nanocrystals
Source: Nat Commun. 2018 Apr 17;9:1518. doi: 10.1038/s41467-018-03965-8 (PMC5904181; doi:10.1038/s41467-018-03965-8)
Supplement: Supplementary file 1 — Supplementary Information [file 41467_2018_3965_MOESM1_ESM.pdf]

Supplementary Information

Resonantly Enhanced Multiple Exciton Generation through Below-Band-Gap Multi-Photon Absorption in Perovskite Nanocrystals

Manzi et al.

Supplementary Note 1. Material characterization

After synthesizing the $CsPbBr_3$ nanocubes (NCs) according to our previously published work,[1] the nanocrystals were drop casted on glass substrates. $CsPbI_3$ NCs were obtained via a subsequent halide ion exchange described in our previous work[2], and large (100 nm) $CsPbBr_3$ NCs were prepared using the same approach reported in ref. 1 but with a decreased ligand amount (0.2 ml oleic acid and 0.2 ml oleylamine instead of 0.5 ml as used there). For the spectroscopic measurements an absorption spectrometer Varian Cary 5000 UV-VISNIR equipped with an integrating sphere setup was used. For photoluminescence (PL) measurements the samples were excited with a monochromated Xe-lamp. PL spectra were taken with a Fluorolog-3 FL3-22 (Horiba Jobin Yvon GmbH) spectrometer equipped with a water-cosoled R928 PMT photomultiplier tube mounted at a 90° angle. The morphology of the samples was investigated using a JEOL JEM-1011 TEM operating at an accelerating voltage of 80-100 kV. For SEM measurements, a Gemini Ultra Plus field emission scanning electron microscope with a nominal resolution of $\sim$2 nm (Zeiss, Germany) was used.

Supplementary Note 2. Experimental setup

The excitation for the MPE-PL measurements was provided by a SuperK EXTREME supercontinuum white light laser with a repetition rate of 78 MHz (NKT Photonics) coupled to an Acusto-optic Tunable Filter (AOTF) to tune the output wavelength in the range between 680 nm and 1080 nm. The laser was focused by a 100x magnification 0.75 numerical

aperture Epiplan-Neofluar Objective (Carl Zeiss Microscopy) to the specimen. The PL from the samples was collected through the same objective to a CCD coupled spectrometer.

Supplementary Note 3. Feasibility studies

The experiments reported in this work involved optical excitation with focused picosecond laser pulses. To check the applicability of our findings in solar applications, we have estimated the degree to which solar light would have to be focused down to reach the photon fluxes obtained with the applied pulsed laser. Considering a laser fluence $\sim 1\ \mu J \cdot cm^{-2}$, which corresponds to the observation of the first resonance in $CsPbBr_3$ assigned to $\lambda_4 = 700\ nm$, and a pulse duration of 15 ps, we obtain a value for the irradiance $I_{\text{laser}} = 6.7 \cdot 10^4\ Wcm^{-2}$. The solar irradiance associated to the same wavelength at sea level, considering the AM 1.5 G spectrum, is about $I_{\text{sun}} = 0.12 \cdot 10^{-3}\ Wcm^{-2}$ for a specific width comparable to our ps laser pulses. Therefore, to obtain the same irradiance used in our experiments, one would need to focus a 1 cm^2 area of solar light down to about 1 μm^2. The diffraction limit at 700 nm defines the minimum spot size to 0.15 μm^2, which is one order of magnitude smaller than the required value, considering a numerical aperture NA = 1.6. This simple estimation shows that the proposed mechanism is feasible for enhancing the efficiency of solar energy conversion through the use of solar concentrators.

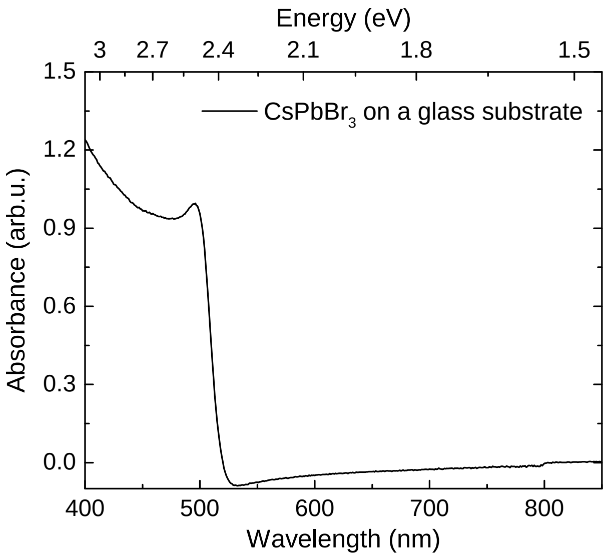

Supplementary Figure 1 One-photon optical absorption of $CsPbBr_3$ NCs on a glass substrate. Integrating sphere absorbance spectra of $CsPbBr_3$ nanocubes on a glass substrate in arbitrary units.

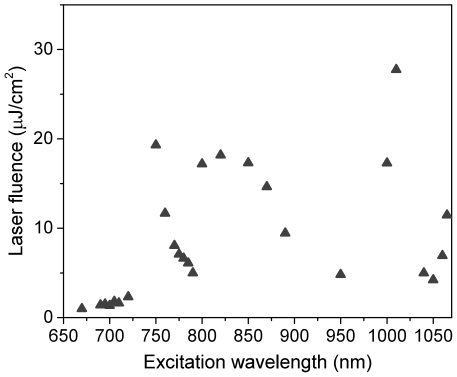

Supplementary Figure 2 Wavelength-dependent laser fluence of the excitiation laser. These values were used for normalizing the PL intensities in Fig. 3a.

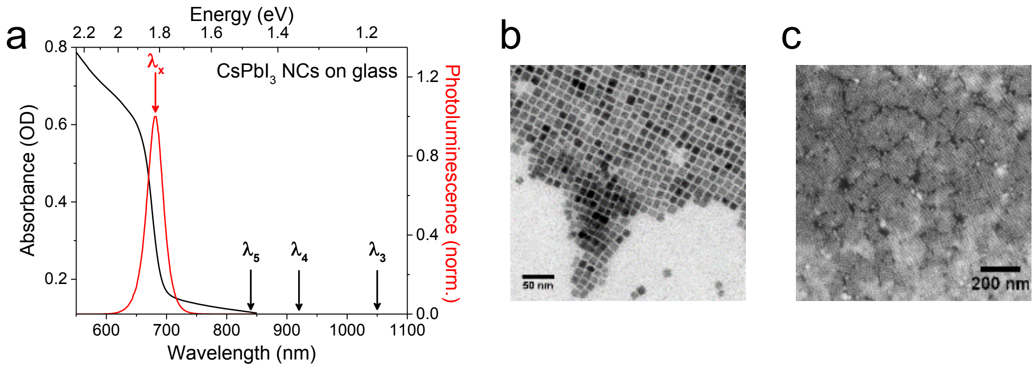

Supplementary Figure 3 Optical and morphological characterization of $CsPbI_3$ nanocubes. **a**, Absorbance (left axis) and photoluminescence spectra (right axis) of $CsPbI_3$ nanocubes on substrate, excitation wavelength 450 nm. The arrows indicate the position of the photoluminescence emission $\lambda_x = 682.5$ nm (corresponding to an exciton energy $E_\text{x} = 1.81$ eV), and the excitation wavelengths λ_5, λ_4 and λ_3 corresponding to the MPE-MEG resonances. **b**, TEM image of $CsPbI_3$ nanocubes, scale bar 50 nm. **c**, SEM image of the $CsPbI_3$ nanocube film, scale bar 200 nm.

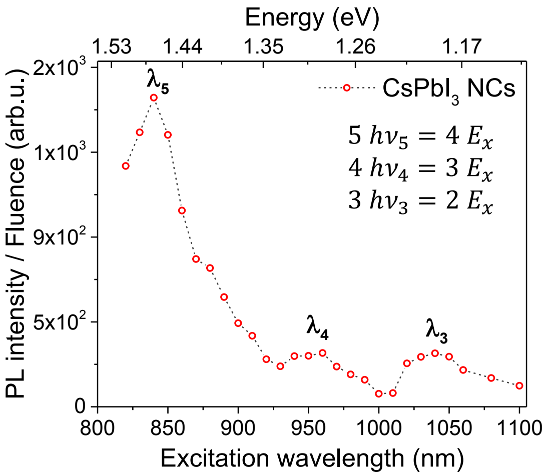

Supplementary Figure 4 Nonlinear absorption-induced PL in $CsPbI_3$ NCs. Photoluminescence intensity of $CsPbI_3$ NCs as a function of the below-band-gap excitation wavelength. The signals are normalized for the corresponding laser fluences. The dashed line is a guide to the eye. The MEG-MPE resonances are centred around $\lambda_5 = 840$ nm, $\lambda_4 = 940$ nm and $\lambda_3 = 1030$ nm, corresponding to photon energies $h\nu_5 = 1.47eV$, $h\nu_4 = 1.32eV$ and $h\nu_3 = 1.20eV$ respectively. These can be interpreted as MEG-MPE resonances satisfying the equations inset in the Figure, with $E_\mathrm{x} = 1.81eV$.

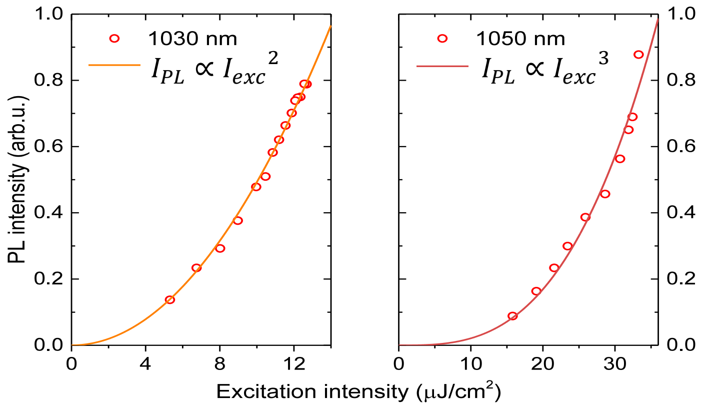

Supplementary Figure 5 Order of the absorption processes for excitation wavelengths around λ_2. Integrated photoluminescence intensity of $CsPbBr_3$ nanocubes as a function of laser excitation intensity relative to the excitation wavelength 1030 nm (left) and 1050 nm (right). The data is fitted with power functions (solid lines). The order of the multiple photon absorption process is 2 for excitation at 1030 nm and 3 for excitation at 1050 nm.

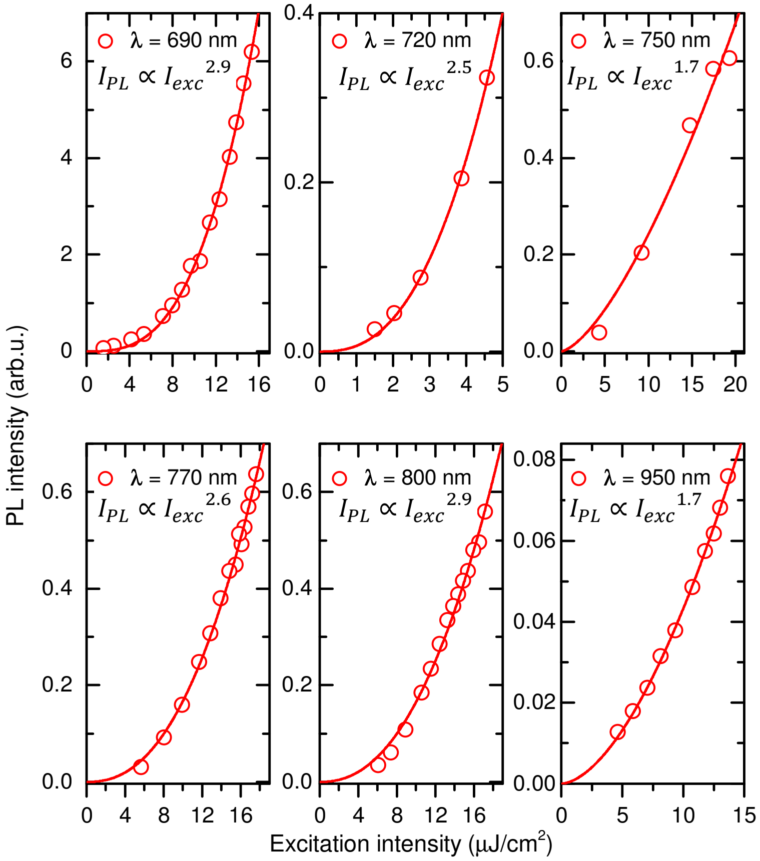

Supplementary Figure 6 Order of the absorption processes for excitation wavelengths around λ_3 and λ_4. Integrated photoluminescence intensity of $CsPbBr_3$ nanocubes as a function of laser excitation intensity relative to the excitation wavelength 690 nm, 720 nm, 750 nm, 770 nm, 800 nm and 950 nm, respectively. The data is fitted with power functions (solid lines), the order of which corresponds to the order of the multiple photon absorption process.

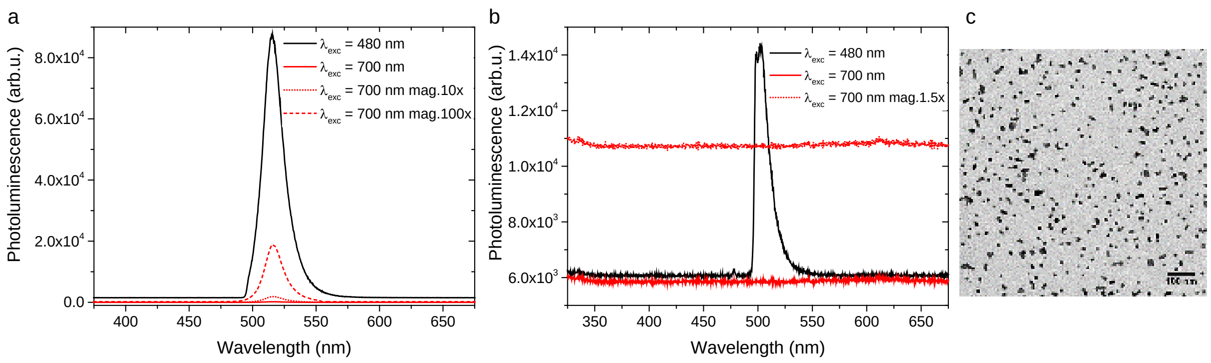

Supplementary Figure 7 1-photon excited photoluminescence *vs.* MPE photoluminescence in $CsPbBr_3$ NCs. PL spectra of $CsPbBr_3$ NCs densely arranged on a substrate (**a**) and of a diluted dispersion of the NCs (**b**). The excitation wavelengths were 480 nm (1-photon excitation) and 700 nm (below-band-gap excitation). **c**, TEM image of the diluted $CsPbBr_3$ NCs, scale bar 100 nm.

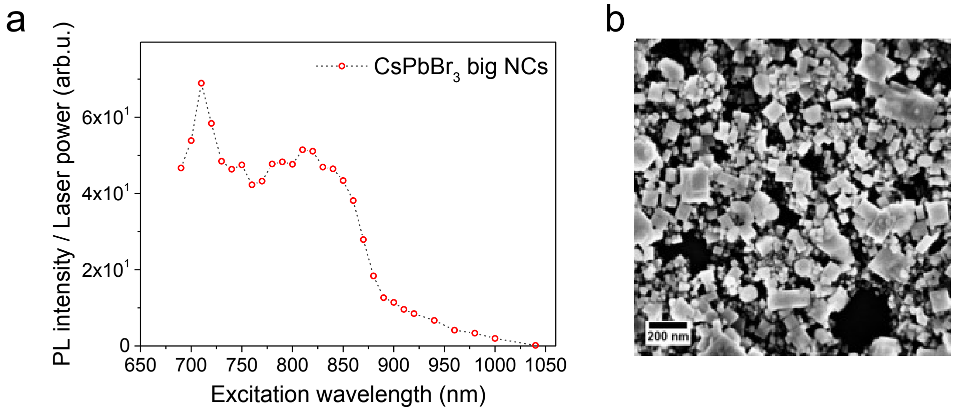

Supplementary Figure 8 Nonlinear absorption-induced PL in large (∼ 100 nm) $CsPbBr_3$ NCs. **a**, Photoluminescence intensity of $CsPbBr_3$ NCs as a function of the below-band-gap excitation wavelength. The intensities are normalized by the laser power. The dashed line is a guide to the eye. **b**, SEM image of the NCs on the substrate, scale bar 200 nm.

Supplementary References

1. Tong, Y. *et al.* Highly Luminescent Cesium Lead Halide Perovskite Nanocrystals with Tunable Composition and Thickness by Ultrasonication. *Angew. Chemie - Int. Ed.* **55**, 13887–13892 (2016).

2. Tong, Y. *et al.* From Precursor Powders to CsPbX3 Perovksite Nanowires: One-pot Synthesis, Growth Mechanism and Oriented Self-assemblies. *Angew. Chemie - Int. Ed.* **56**, 13887–13892 (2017).
